# Environment Monitoring for Anomaly Detection System Using Smartphones †

**DOI:** 10.3390/s19183834

**Published:** 2019-09-05

**Authors:** Van Khang Nguyen, Éric Renault, Ruben Milocco

**Affiliations:** 1 SAMOVAR, CNRS, Télécom SudParis, Institut Polytechnique de Paris, 91011 Évry, France; 2 College of Education, Hue University, 530000 Hue, Vietnam; 3 GCAyS, UNComahue, Buenos Aires 1400, 8300 Neuquén, Argentina

**Keywords:** anomaly detection, anomalies aggregation, smartphone sensing, sensor networks

## Abstract

Currently, the popularity of smartphones with networking capabilities equipped with various sensors and the low cost of the Internet have opened up great opportunities for the use of smartphones for sensing systems. One of the most popular applications is the monitoring and the detection of anomalies in the environment. In this article, we propose to enhance classic road anomaly detection methods using the Grubbs test on a sliding window to make it adaptive to the local characteristics of the road. This allows more precision in the detection of potholes and also building algorithms that consume less resources on smartphones and adapt better to real conditions by applying statistical outlier tests on current threshold-based anomaly detection methods. We also include a clustering algorithm and a mean shift-based algorithm to aggregate reported anomalies on data to the server. Experiments and simulations allow us to confirm the effectiveness of the proposed methods.

## 1. Introduction

The mobile phone sensor system is promising great potential for applications. For the past few years, smartphones have become more popular and powerful. Any smartphone today contains many different sensors that sense information from the surrounding environment such as a camera, a microphone, a GPS sensor, an accelerometer, a proximity sensor, an ambient light sensor, a magnetometer, a barometer, an air humidity sensor, a thermometer, etc. Besides, network costs are getting lower and lower. Therefore, the research and application of the mobile phone sensor system has become increasingly popular.

Many studies of smartphone sensors and mobile phone sensor system have been described in the literature [1,2,3]. Common areas of application are: personal health monitoring, calculation of environmental impact, fall detection, freezing of gait detection, monitoring and traffic conditions, monitoring noise and ambiance, etc.

It is possible to classify areas such as monitoring road and traffic conditions, monitoring noise, and ambiance and fall detection into monitoring and anomaly detection systems. In the future, there will be many other applications that can be exploited from monitoring and anomaly detection systems based on data from sensors such as ambient light sensors, magnetometers, barometers, air humidity sensors, and thermometers. The basic processing of monitoring and anomaly detection systems is as follows: Data from sensors are collected regularly in time. These data on smartphones are comprised of anomalies such as road anomalies, falls, or unusual sounds. If it is necessary to compute environmental conditions, such as road flatness or noise, depending on the type of applications, all data or only the remaining part of data is analyzed. All final required results are sent to the server for further processing. This paper presents the overall architecture of the system we developed for the monitoring and the detection of anomalies. With this model, we propose lightweight algorithms to identify anomalies on smartphones and an algorithm to aggregate these anomalies and the associated data on the server. We have also conducted experiments to validate the approach and evaluate the results of the proposed algorithms. To evaluate our road anomaly detection algorithms, we applied them during experiments on real data. For the specific case of the anomaly aggregation algorithms used to decide whether anomalies are true potholes or artifacts, we performed simulations to evaluate the results because it was not possible to get enough real data for each pothole.

The rest of the paper is organized as follows: Section 2 introduces our smartphone-server architecture for environment anomaly detection. Section 3 presents the way to apply the Grubbs test on threshold-based anomaly detection algorithms and experiments to verify this method. Section 4 describes the problem of anomaly data aggregation, our solution, and the simulation method. Finally, Section 5 concludes the paper.

## 2. System Architecture

**Figure 1:**
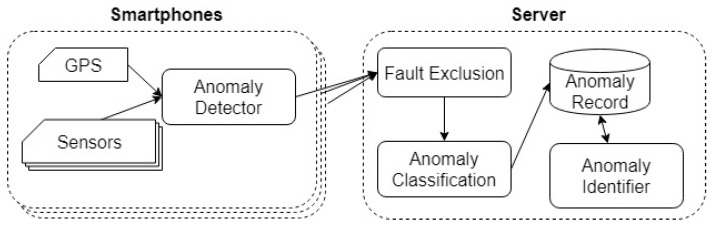
Smartphone-server system architecture for road anomaly detection, © 2019 IEEE [4].

In our anomaly detection system, we divided the anomaly detection task into several stages. Some of these stages are handled at the server to reduce the power consumption of smartphones (see Figure 1). The functionality of the system components are described as follows.

- The Anomaly Detector aims at initially detecting anomalies with a lightweight algorithm, i.e., an algorithm that consumes very few device resources. A resident program is installed on the phone to read the data from the accelerometer and the GPS sensor. These data are passed to the anomaly detection component under a certain condition, for example when the smartphone reaches a speed greater than a certain value for the case of the monitoring and traffic conditions. Then, the program receives a list of anomalies to send to the server when connected.
- The Fault Exclusion component is intended to eliminate false anomalies caused by user’s actions. This component can be viewed as a detached part of the Anomaly Detector. The separation between the two components (Anomaly Detector and Fault Exclusion) is intended to distribute processing between the client and the server to optimize resource consumption of the smartphones.
- The Anomaly Classification component classifies anomalies, which also allows for the elimination of less reliable anomalies.
- The Anomaly Identifier component aggregates anomaly reports from multiple smartphones to locate anomalies and compute a confidence weight associated with each location.

In this paper, we focus on two important components: the Anomaly Detector and Anomaly Identifier.

## 3. Anomaly Detection Algorithm

Many different types of anomaly detection methods have been proposed, depending on the purpose and type of sensor data used. In this section, we propose an improvement for road anomaly detection methods based on acceleration data, also known as vibration-based methods.

### 3.1. Related Works

Current vibration-based methods for road anomaly detection using smartphones can be divided into two classes: threshold-based methods and classification-based methods [5]. Threshold-based methods are simple algorithms to identify potholes by verifying if a component or a value derived from a function of a measured acceleration exceeds a specific threshold. With the pothole patrol system, Jakob Eriksson et al. [6] proposed an algorithm based on a set of threshold-based filters. Prashanth Mohan et al. [7] proposed the the Nericell road monitoring system in which they detected braking by comparing the average value of the *x* component of the acceleration (the projection on the axis in the direction of the motion) in a sliding window to a threshold. They detected bumps and potholes by comparing the *z* component of the acceleration (the projection on the vertical axis) to two threshold values depending on the speed. Astarita Vittorio et al. [8] proposed to compare the distance between two extreme acceleration values (min and max) with a threshold to detect road anomalies. Artis Mednis et al. [9] proposed four threshold-based algorithms Z-THRESH, Z-DIFF, STDEV(Z), G-ZERO (see below). In classification-based methods, several features such as the mean, variance, and root mean square of the three axes, as well as the correlation between the axes [10,11,12] are first extracted from the acceleration data. Second, a machine-learning algorithm typically using Support Vector Machines (SVM) is applied to classify these features into road anomaly or artifact. We found that the current methods were less adaptable to real conditions such as the flatness of the road, the type of vehicle, the speed of travel, the quality of the suspension, and smartphone type. For the threshold-based approach, the authors used a fixed threshold. Meanwhile, the fluctuation of acceleration data depended very much on real conditions. For classification-based methods, training was performed on a road segment with a vehicle with some characteristics. It was difficult to produce good results in this study when applied to vehicles with other characteristics.

We investigated how to improve threshold-based algorithms to achieve small complexity algorithms and the least phone resource consumption. We chose threshold-based algorithms because they require low resources, while classification-based algorithms require many resources, leading to much energy consumption. Obviously, the smartphone battery power must be reserved for user functions.

Here are the algorithms that we improved and on which we did experiments to validate their effectiveness:

- Four algorithms proposed by Artis Mednis et al. [9]:
  - **Z-THRESH**: If the amplitude of the value on the *z*-t axis of acceleration data is greater than a specified threshold, a road anomaly is detected.
  - With **Z-DIFF**, events are detected when the difference between two consecutive values is greater than a specific threshold.
  - **STDEV(Z)** is based on the standard deviation in a sliding window. When the standard deviation is greater than a specific threshold, an event is detected.
  - With **G-ZERO**, an event is detected when the values of all three axes are less than a specific threshold.
- The algorithm proposed by Vittorio et al. [8] uses the vertical acceleration provided by both the accelerometer and GPS sensor only. For simplicity, we refer to this algorithm as DVA-THRESH in the rest of this paper. Since the GPS data frequency was 1 Hz and one of the accelerometers was at least 5 Hz, the authors preprocessed the accelerometer data in groups of one second by computing $a_{z\_min}$, $a_{z\_max}$, and $a_{z\_avg}$. The detection was based on the difference in vertical acceleration impulse defined by $DVA=a_{z\_max}-a_{z\_min}$.
  The road anomaly filter is given by the following operation:
  $$DVA=\left\{\;\begin{array}{cc} 0 & \mathrm{if}\;DVA\leq {DVA}^{\mathrm{th}}\\ DVA & \mathrm{if}\;DVA>{DVA}^{\mathrm{th}} \end{array}\right.$$
  where $DVA^{\mathrm{th}}$ is a reference set of DVA values that was determined by previous experiments.

### 3.2. Improvement of Anomaly Detection Algorithms

We considered the road anomaly through the abnormal sensor data we obtained and compared them to surrounding data. When the car enters a road anomaly like a pothole, the statistic moments of acceleration change. This is the basis for anomaly detection. However, the change of acceleration data depends on many factors. If a fixed threshold value is used, it is not possible to determine the abnormality correctly. “Abnormal data” here must be understood as abnormal when compared to the data before and after the car enters the pothole.

On this basis, we propose an anomaly detection on the small sample of sensor data lastly obtained by using a statistical outlier test method to find anomalies.

We chose to apply the Grubbs test method. This is a very popular outlier test method for univariate datasets. In particular, the application of this method allows us to reduce the computational complexity.

#### Application of the Grubbs’ Test to Threshold-Based Anomaly Detection Algorithms

The Grubbs’ test [13] is a statistical test used to detect outliers in a univariate dataset. The test assumes that the underlying data distribution is normal. Grubbs’ test is defined when the following hypotheses are true:

- $H_{0}$: There is no outlier in the dataset.
- $H_{1}$: There is exactly one outlier in the dataset

The Grubbs’ test statistic is defined as follows:

$$C=\frac{max_{i}\left|x_{i}-\bar{x}\right|}{\sigma }$$

where $\bar{x}$ and σ denote the sample mean and the standard deviation, respectively. For the two-sided test, the hypothesis of no outlier was rejected at the significance level α if:

$$C>G_{N,\alpha }=\frac{N-1}{\sqrt{N}}\sqrt{\frac{t_{\alpha /(2N),N-2}^{2}}{N-2+t_{\alpha /(2N),N-2}^{2}}}$$

where $t_{\alpha /(2N),N-2}^{2}$ denotes the upper critical value of the *t*-distribution with N−2 degrees of freedom and a significant level of α/(2N). For the one-sided test, α/(2N) is replaced by α/N [14].

In order to apply the Grubbs test to the five algorithms mentioned above (namely Z-THRESH, Z-DIFF, STDEV(Z), G-ZERO, and DVA-THRESH), we compared the components of the acceleration data according to the Grubbs formula instead of comparing it with a fixed threshold. Let the data obtained from the acceleration sensor be a sequence denoted as $<t^{i},a_{x}^{i},a_{y}^{i},a_{z}^{i}>$ where $t^{i}$ is the time of sensing and $a_{x}^{i},a_{y}^{i},a_{z}^{i}$ are the three components of the acceleration vector at time $t^{i}$. The value to compare depends on the specific algorithm, and the last *N* comparison values are retained as sample *X* (see Table 1).

We propose to process sensor data as a continuous sequence. Whenever a new acceleration data value is received, sample *X* is updated by adding the new value and removing the oldest one. At the same time, the mean value $\bar{X}$ and standard deviation $\sigma_{X}$ must also be recomputed.

To reduce the complexity of the algorithm, we implemented the update of $\bar{X}$ and $\sigma_{X}$ according to the cumulative method. Suppose *X* is updated by adding $X_{new}$ (the new value in the sliding window) and deleting $X_{old}$ (the oldest value in the sliding window), as $\sigma =\sqrt{\bar{X_{i}^{2}}-{\bar{X}}^{2}}$ (where $\bar{X}$ is the average of the value in *X* and $\bar{X^{2}}$ is the average of the squares of the values in *X*); one just needs to update $\bar{X}$ and $\bar{X^{2}}$ as follows:

$${\bar{X}}_{new}={\bar{X}}_{old}+\frac{X_{new}-X_{old}}{N}$$

$${\bar{X^{2}}}_{new}={\bar{X^{2}}}_{old}+\frac{X_{new}^{2}-X_{old}^{2}}{N}$$

### 3.3. Experiment

#### 3.3.1. Collection and Adjustment of Data

We collected accelerometer and GPS data on nearly 40 km of road in Hue city using both a Mazda 3 car and a Honda Lead 125 scooter. The smartphone used was a Wiko WIM Lite equipped with a 15.38-Hz accelerometer. We developed a program for the phone that allowed saving sensor data as a sequence of tuples like:

<time, 3-axis acceleration>
<time, location, speed>

To build the ground truth, we added a data collection application of buttons to allow marking every time the vehicle entered a road anomaly. There were 217 anomalies marked with the car and 219 anomalies marked with the scooter. We also built a software program to adjust the ground truth on the computers after data collection. The road anomalies were marked according to the timeline. Marking operations may be slower than the actual time the vehicle enters the anomaly. Therefore, we needed to adjust them manually to ensure accuracy.

#### 3.3.2. Experiment Process and Results

We installed the five algorithms Z-THRESH, Z-DIFF, STDEV(Z), G-ZERO, and DVA-THRESH on the smartphone to test them. Each algorithm had two versions installed: the original version and the improved version with the Grubbs test method. Acceleration data were processed as a continuous data sequence, to simulate a real-time anomaly detection. The size of sample *N* in our experiment was 100. When an anomaly was detected, the corresponding data segment was compared to the ground truth data based on time. If there was an anomaly there, this was a True Positive (TP); otherwise, it was a False Positive (FP). A non-detected anomaly in the ground truths was a False Negative (FN).

In this experiment, the number of detected negative conditions, also known as True Negative (TN), cannot be determined. Note that data were processed as a continuous sequence; they were not cut into windows. Therefore, the number of negative cases was uncountable.

We chose the evaluation method based on the Precision (*P*) and Recall (*R*) parameters as defined by:

$$P=\frac{TP}{TP+FP}\;\mathrm{and}\;R=\frac{TP}{TP+FN}$$

Unlike methods such as the Receiver Operating Characteristic (ROC) curve [15], methods based on precision and recall are computed without the need for true negative values [16]. The experimental results were evaluated through the precision-recall curve and the F-measure index (also known as the $F_{1}$ score), which is the harmonic mean of both precision and recall:

$$F_{1}=2\times \frac{P\times R}{P+R}$$

**Figure 2:**
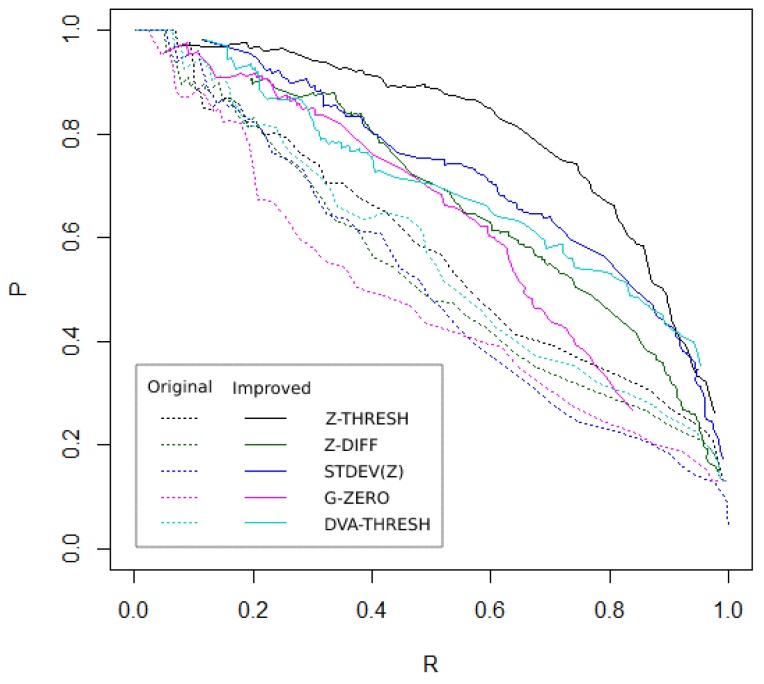
The precision-recall curve graphs, © 2019 IEEE [4].

The analysis of the results on the precision-recall curves showed that the algorithms were providing better results when improved with the Grubbs test method. Figure 2 shows the corresponding precision-recall curves when applying the above five algorithms, both the original version and the improved version, to all data collected. Ideally, if there is no error, *P* and *R* should be equal to 1, i.e., the closer to the upper-right corner, the better the result. As shown in Figure 2, the graphs of the improved algorithms were all above, closer to the upper-right corner than the the original algorithms.

Experimental results with the Z-THRESH algorithm on the three different datasets (the data collected by cars, the data collected by the scooter, and the aggregated data) showed that not only did it provide better results, but the improved algorithm also showed more stable results when changing vehicles. This proved that the application of the Grubbs test allowed the algorithm to adapt well to real conditions because the amplitude of the acceleration data when using cars and scooters was very different.

**Figure 3:**
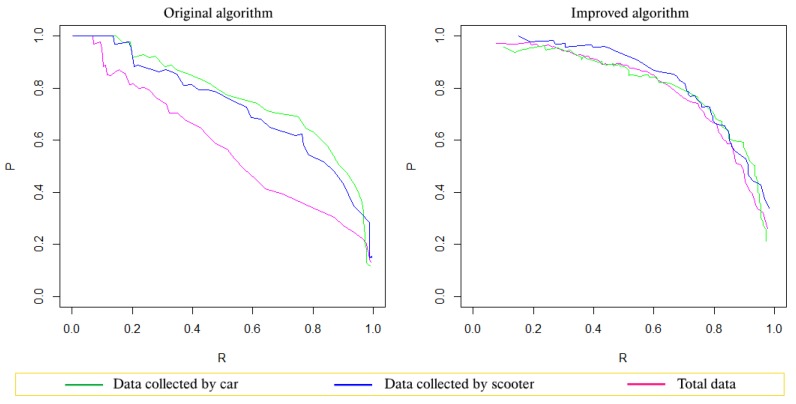
The precision-recall curves graphs of the Z-THRESH algorithm on the three datasets ©, 2019 IEEE [4].

**Figure 4:**
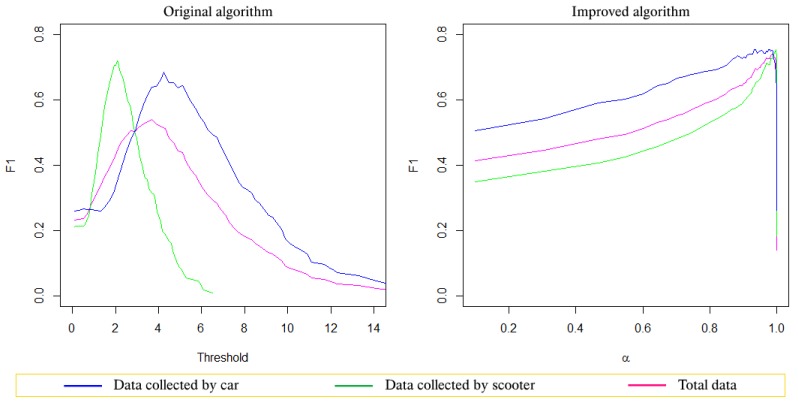
The F-measure graphs of the Z-THRESH algorithm on the three datasets, © 2019 IEEE [4].

The Precision-recall curves in Figure 3 show that the original Z-THRESH algorithm resulted in a significant reduction of the precision when the data became diverse. Meanwhile, the improved algorithm gave good and very stable results, as all three curves were close to each other. Analyzing the F-measure graph (see Figure 4), one can also see that with the original Z-THRESH algorithm, the F-measure reached a maximum at very different threshold values when testing on datasets. In contrast, with the improved algorithm, not only the maximum value of the F-measure was greater, but more importantly, the F-measure reached a similar maximum value even when it was applied to different datasets.

We applied and tested the application of test outliers on the detection of road anomalies. The results showed that this approach achieved very good results. The principle of finding anomalies compared to the surrounding data can also be applied to other types of data and other applications. For example, the application of a fall detection on acceleration data also has approaches based on a threshold [17] that can be improved.

## 4. Anomaly Identifier

When multiple anomalies are detected and sent to the center from multiple smartphones, an anomaly can be reported several times. The position of an anomaly was reported determined by GPS, and there were some errors. The problem was aggregating that data to determine the more accurate location and reliability of the information about the anomaly.

There have been some research works discussing anomaly data aggregation methods. Notably, Jakob Eriksson et al. [6] proposed a clustering method whereby two anomalies were grouped in a cluster if their distance was less than a given value Δd. However, the maximum distance between anomalies in a cluster was limited by a given value Δt. According to the authors, an event was valid if there was at least *k* elements in the cluster to which it belonged. Zhaojian Li et al. [18] proposed a clustering method for grouping anomalies based on the Mahalanobis distance [19]. The main idea for clustering was as follows: an event with coordinates *x* was set in cluster *C* if the Mahalanobis distance D(x,C) was less than a given *p* value. The Mahalanobis distance was computed using the equation:

$$D\left(x,C\right)=\sqrt{{\left(x-\mu_{C}\right)}^{T}\Sigma_{C}^{-1}\left(x-\mu_{C}\right)}$$

where $\mu_{C}$ is the weighted mean and $\Sigma_{C}$ is the weighted covariance matrix of *C*. The authors proposed to use the event time as a parameter to compute $\mu_{C}$ and $\Sigma_{C}$, i.e.,

$$\mu_{C}=\frac{\sum_{k=1}^{K_{C}}f\left(t-t_{Ck}\right)x_{Ck}}{\sum_{k=1}^{K_{C}}f(t-t_{Ck})}$$

$$\Sigma_{C}=\frac{\sum_{k=1}^{K_{C}}f\left(t-t_{Ck}\right)\left(x_{Ck}-\mu_{C}\right){\left(x_{Ck}-\mu_{C}\right)}^{T}}{\sum_{k=1}^{K_{C}}f(t-t_{Ck})}$$

where $t_{Ck}$ and $x_{Ck}$ are the time and coordinates of data point $k^{\mathrm{th}}$ in cluster *C* and *t* is the current time. Function *f* is an exponential function of the form $f\left(\tau \right)=\alpha^{-\lambda \tau }$ where α>1 and λ>0 are two positive scalars.

The common disadvantage of the above two methods was that they only stopped at the clustering, not yet calculating the position of anomalies, as well as the lack of reliability assessment. In addition, Jakob Eriksson’s method was unclear about how to remove the anomalies from a cluster if the maximum distance in the cluster exceeded the allowed value. Therefore, we propose a method of anomaly position aggregation in which the main part is the mean shift-based algorithm.

The position of a reported anomaly is a GPS location with horizontal accuracy. GPS location data can be considered Gaussian distributed around the real position [20,21,22], and the horizontal accuracy of the GPS on the smartphone is the RMS (Root Mean Squared) accuracy in two dimensions [22], i.e.,

$$RMS=\sqrt{\sigma_{x}^{2}+\sigma_{y}^{2}}$$

where $\sigma_{x}^{2}$ and $\sigma_{y}^{2}$ are the variance according to the axes. As GPS data on smartphones do not contain specific $\sigma_{x}$ and $\sigma_{y}$ values, one can approximate $\sigma_{x}$ and $\sigma_{y}$ to *accuracy*$/\sqrt{2}$. In the scope of this study, we used a variance value $\sigma^{2}=\sigma_{x}^{2}=\sigma_{y}^{2}$ for each data point. In addition, the calculation of the GPS error was based on the assumption that the components of the error were independent [23], so we assumed that the GPS position on the plane was distributed according to the Gaussian distribution with the covariance matrix $\Sigma =\mathrm{diag}(\sigma^{2},\sigma^{2})$. In practice, the type of GPS accuracy should be determined as a parameter to adjust the way of computing the variance.

The anomaly locations looked like the observations of a bi-variate Gaussian mixture model in which variances corresponding to each observation point were known. Our solution was to use the mean shift method to find the highest points of the probability density estimation function. However, as the location of the anomalies is scattered throughout the world, we first needed to split the data into non-interconnected clusters, to increase accuracy and reduce computation time for the mean shift-based algorithm.

### 4.1. Simple Clustering

This clustering is a pretreatment to divide the reported anomalies into isolated regions. Assuming $p_{i}$ and $p_{j}$ are two points corresponding to reported anomalies, these two points are referred to as *far apart* if the distance between them is greater than a specific value $d_{ij}$:

$$\mathrm{far}\left(p_{i},p_{j}\right)=\left\{\;\begin{array}{cc} \mathrm{true} & \mathrm{if}∥p_{i}-p_{j}∥>d_{ij}\\ \mathrm{false} & \mathrm{otherwise} \end{array}\right.$$

where $\sigma_{i}$ and $\sigma_{j}$ are the standard deviations associated with points $p_{i}$ and $p_{j}$, respectively. Then, let function include(C,p) used to determine if point *p* belongs to cluster *C* be defined by:

$$\mathrm{include}\left(C,p\right)=\left\{\;\begin{array}{cc} \mathrm{true} & \mathrm{if}\;\exists \;q\in C/\mathrm{far}(p,q)=\mathrm{false}\\ \mathrm{false} & \mathrm{otherwise} \end{array}\right.$$

The value $d_{ij}$ needs to be chosen large enough so that in the case where $p_{i}$ and $p_{j}$ are not in the same cluster, the probability that these two points belong to the same real anomaly is very small. Note that under the Gaussian distribution assumption mentioned earlier, the probability of an observation falling outside the circle with radius *r* and the center being the anomaly position is $\exp\left(-\frac{r^{2}}{2\sigma^{2}}\right)$, where $\sigma^{2}$ is the variance. Suppose the number of observations is *n*, then the probability of having exactly one observation falling outside this circle, according to the binomial distribution, is $C_{n}^{1}\exp{\left(-\frac{r^{2}}{2\sigma^{2}}\right)}^{1}{\left(1-\exp\left(-\frac{r^{2}}{2\sigma^{2}}\right)\right)}^{n-1}<n*\exp\left(-\frac{r^{2}}{2\sigma^{2}}\right)$. Hence, if $p_{i}-p_{j}>d_{ij}$ and $p_{j}\in C$, the probability of $p_{i}$ belonging to an anomaly of cluster *C* will be less than $\delta =N_{max}*\exp\left(-\frac{d_{ij}^{2}}{2\sigma_{i}^{2}}\right)$, where $N_{max}$ is the maximum number of reports possible for an anomaly. The value $d_{ij}$ can be selected based on the desired value of δ. In fact, we also need to ensure that $p_{j}$ does not belong to an anomaly of the cluster of $p_{i}$. Therefore, we propose the following formula:

(1) $$d_{ij}=\max\left(\sigma_{i},\sigma_{j}\right)\sqrt{-2\ln\left(\frac{\delta }{N_{\max}}\right)}$$

For example, if $N_{max}=$ 10,000, δ=0.0001, then $d_{ij}\approx 6*max\left(\sigma_{i},\sigma_{j}\right)$. The value $N_{max}$ can be predicted from reported data. Observe that it is updated, but does not grow infinitely over time since the system removes old reports periodically.

Formula (1) only ensures that the value of $d_{ij}$ is “large enough”, not optimal for clustering. The goal is for this simple clustering to be performed quickly in a cumulative manner. More detailed clustering technique is addressed in Section 4.2.

**Algorithm 1** Update clusters.**INPUT:**$C(C_{1},C_{2},\cdots ,C_{K})$ {current cluster list}, *p* {new data point}**OUTPUT:***C* {new cluster list}  C^*^ ← ∅ {C^*^ is to contain all current clusters that *p* belongs to}  **for**
$k\in \left\{1,\cdots ,K\right\}$
**do**   **if**
$include(C_{k},p)$
**then**    $C^{*}\leftarrow C^{*}\cup \left\{C_{k}\right\}$   **end if**  **end for**  {Next, take the union of all clusters in C^*^ and add *p* to get the new cluster}  *C* ← *C*/C^*^  $C_{new}\leftarrow \left(\overset{|C^{*}|}{\underset{i=1}{⋃}}C_{i}^{*}\right)\cup \left\{p\right\}$  $C\leftarrow C\cup \left\{C_{new}\right\}$

The data clustering algorithm is executed cumulatively, i.e., it is called when a new data point is added. The algorithm to update the cluster is presented in Algorithm 1.

### 4.2. Mean Shift-Based Algorithm to Find Anomaly Positions

The next step after applying the simple clustering method presented above is to find the local maxima of the probability density (modes) based on the reported anomaly positions and the associated GPS accuracy. A mode is the most likely point where anomalies have been detected. Given the characteristic of the reported anomalies data, we found that the *variable bandwidth mean shift* method with the *Gaussian kernel* was an effective option to solve the problem. Dorin Comaniciu showed that mean shift to variable bandwidth is an adaptive estimator of the normalized gradient of the underlying density [24]. Carreira-Perpinan and Miguel A also confirmed that, when the kernel was Gaussian, mean shift was an expectation-maximization algorithm [25]. As far as the problem of the aggregation of anomalies is concerned, it can be seen that it was a Gaussian mixture model. Computations via this model can be quite complicated, and each observation corresponds to a different variance. Nevertheless, this complexity can be handled by a variable bandwidth mean shift in which each data point is combined with a bandwidth value. Moreover, the mean shift algorithm solves two problems simultaneously: the mode-finding problem to locate anomalies and the clustering problem to calculate the reliability of the identified modes.

Let $p_{i}$, i=1…n be a set of points in the $R^{d}$ space and the associated bandwidth $h_{i}$, the density estimator computed at a given point *p* with kernel profile k(p) is given by [26]:

(2) $$\overset{\wedge }{f}\left(p\right)=\frac{1}{n}\sum_{i=1}^{n}\frac{1}{h_{i}^{d}}k\left({∥\frac{p-p_{i}}{h_{i}}∥}^{2}\right)$$

The local maximum of the density function that can be iteratively reached using a mean shift vector is given by [26]:

(3) $$m\left(p\right)=\frac{\sum_{i=1}^{n}\frac{p_{i}}{h_{i}^{d+2}}g\left({∥\frac{p-p_{i}}{h_{i}}∥}^{2}\right)}{\sum_{i=1}^{n}\frac{1}{h_{i}^{d+2}}g\left({∥\frac{p-p_{i}}{h_{i}}∥}^{2}\right)}-p$$

where $g\left(x\right)=-k^{\prime }\left(x\right)$. We use the multivariate Gaussian kernel:

(4) $$k\left(x\right)=\exp\left(-\frac{1}{2}x\right)$$

so that:

(5) $$g\left(x\right)=-\frac{1}{2}\exp\left(-\frac{1}{2}x\right)$$

In this study, each point $p_{i}$ was considered an observation of a Gaussian distribution with the associated standard deviation $\sigma_{i}$. We propose to use bandwidth $h_{i}$ = $\lambda \sigma_{i}$, with λ being an experimental parameter adjusted around one to get the best results for each situation. The anomalies were considered in the plane, and after replacing d=2, $h_{i}$ = $\lambda \sigma_{i}$ and Equation (5) in Equation (3), we get:

(6) $$m\left(p\right)=\frac{\sum_{i=1}^{n}\frac{p_{i}}{\lambda^{4}\sigma_{i}^{4}}\exp\left(-\frac{1}{2}{∥\frac{p-p_{i}}{\lambda \sigma_{i}}∥}^{2}\right)}{\sum_{i=1}^{n}\frac{1}{\lambda^{4}\sigma_{i}^{4}}\exp\left(-\frac{1}{2}{∥\frac{p-p_{i}}{\lambda \sigma_{i}}∥}^{2}\right)}-p$$

Based on the mean shift vector, we developed an algorithm that is similar to the one presented in [27] to find the maximum density points (see Algorithm 2). The algorithm assigns a weight for every maximum point found. These weighted points are stored in the system to assess reliability. We dealt with it as an anomaly if the associated weight was greater than a given threshold. It is also possible to improve the algorithm by eliminating mode points that follow some weight-related criteria.

**Algorithm 2** Anomaly identification.
**INPUT:**
 $p_{1},p_{2},\ldots ,p_{n}$ {list of data points} $\sigma_{1},\sigma_{2},\ldots ,\sigma_{n}$ {list of standard deviations corresponding to points} λ {bandwidth parameter} ε, ρ {error parameters}**OUTPUT:***Q*, *w* {list of modes and list of weights} *Q* ← ∅ **for**
$i\in \left\{1,\ldots ,n\right\}$
**do**  $p\leftarrow p_{i}$  **repeat**   $m\left(p\right)\leftarrow \frac{\sum_{j=1}^{n}\frac{p_{j}}{\lambda^{4}\sigma_{j}^{4}}\exp\left(-\frac{1}{2}{∥\frac{p-p_{j}}{\lambda \sigma_{j}}∥}^{2}\right)}{\sum_{j=1}^{n}\frac{1}{\lambda^{4}\sigma_{j}^{4}}\exp\left(-\frac{1}{2}{∥\frac{p-p_{j}}{\lambda \sigma_{j}}∥}^{2}\right)}-p$   p←p+m(p)  **until**
$\left|m(p)\right|<\varepsilon$  q=nearestPoint(Q,p) {*nearestPoint(Q,p)* returns a point in Q that is closest to p}  **if**
∃q
**and**
$∥q-p∥<\rho$
**then**   $w\left(q\right)\leftarrow w\left(q\right)+\frac{1}{\sigma_{i}^{2}}\exp\left(-\frac{{∥q-p_{i}∥}^{2}}{2\sigma_{i}^{2}}\right)$  **else**   $w\left(p\right)\leftarrow \frac{1}{\sigma_{i}^{2}}\exp\left(-\frac{{∥p-p_{i}∥}^{2}}{2\sigma_{i}^{2}}\right)$   $Q\leftarrow Q\cup \left\{p\right\}$  **end if** **end for**

We recommend using weight function w(p) as a proportion of $\overset{\wedge }{f}\left(p\right)$ with bandwidth $\sigma_{i}$:

(7) $$w\left(p\right)=\sum_{i=1}^{n}\frac{1}{\sigma_{i}^{2}}\exp\left(-\frac{{∥p-p_{i}∥}^{2}}{2\sigma_{i}^{2}}\right)$$

To understand the properties of function *w*, we first consider the function *v* as follows:

(8) $$v\left(p\right)=\sum_{i=1}^{n}\exp\left(-\frac{{∥p-p_{i}∥}^{2}}{2\sigma_{i}^{2}}\right)$$

Assume there is an anomaly at point p(0,0) and there is an observation of the anomaly at point q(x,y). According to the Gaussian distribution with variance $\sigma_{x}^{2}=\sigma_{y}^{2}=\sigma^{2}$, the expected value of the v(p) functions is given by:

(9) $$\begin{array}{cc} E[v(p)] & =\int_{-\infty }^{+\infty }\int_{-\infty }^{+\infty }v(p)f\left(x,y\right)dxdy=\int_{-\infty }^{+\infty }\int_{-\infty }^{+\infty }\exp\left(-\frac{x^{2}+y^{2}}{2\sigma^{2}}\right)\frac{1}{2\pi \sigma^{2}}\exp\left(-\frac{x^{2}+y^{2}}{2\sigma^{2}}\right)dxdy\;\\ & =\frac{1}{2\pi \sigma^{2}}\int_{-\infty }^{+\infty }\int_{-\infty }^{+\infty }\exp\left(-\frac{x^{2}+y^{2}}{2\sigma^{2}}\right)dxdy \end{array}$$

where f(x,y) is the probability density function of a bivariate normal distribution in the case of independent *x* and *y*. The integral in Equation (9) can be computed in polar coordinates, i.e.:

$$\int_{-\infty }^{+\infty }\int_{-\infty }^{+\infty }\exp\left(-\frac{x^{2}+y^{2}}{\sigma^{2}}\right)dxdy=\int_{0}^{+\infty }\int_{0}^{2\pi }\exp(-\frac{r^{2}}{\sigma^{2}})\;r\;dr\;d\theta =\pi \sigma^{2}$$

This leads to an expected value of function *v* equal to:

(10) $$E[v(p)]=\frac{1}{2}$$

From Equations (7), (8), and (10), the expected value of function *w* is:

(11) $$E[w(p)]=\sum_{i=1}^{n}\frac{E[v(p)]}{\sigma_{i}^{2}}=\sum_{i=1}^{n}\frac{1}{2\sigma_{i}^{2}}=\frac{n}{2}\left(\frac{1}{n}\sum_{i=1}^{n}\frac{1}{\sigma_{i}^{2}}\right)=n\frac{\bar{\left(\sigma_{i}^{-2}\right)}}{2}$$

Equation (11) shows that the expected value of the function *w* is directly proportional to the number of reports. Moreover, the smaller the reporting error, the bigger the value in *w*. As a result, function *w* can be used as a weighted function to evaluate the reliability of an anomaly.

Note that Equation (11) applies only for the case of one anomaly. In practice, there might be multiple anomalies, giving rise to several modes. Only points in the *attraction basin* of a mode are computed in the weight function of that mode. The attraction basin of a mode is the largest region in which all the points have trajectories (defined by the mean shift vector) that lead to this mode [28].

In Algorithm 2, two location error parameters (ε and ρ) are used. The repeat ... until loop stops when the mean shift vector is smaller than a positive tolerance [27]. Tolerance value ε is chosen according to the specific application. The greater ε, the faster the program. However, the accuracy of the location of the modes is reduced. With this stop condition, the program can give several results around a real mode. To fix this, a ρ parameter is added. The algorithm must ensure:

(1) the distance between any two modes is greater than or equal to ρ;
(2) if a potential point is removed, there must be at least one other convergence point selected as the mode so that the distance between them is smaller than ρ.

The ρ parameter must be large enough to ensure that the program only produces one point for each mode, and it must be small enough to prevent different real modes from merging into a single one. In our simulation experiments, we first chose ε and then did an experiment to choose a suitable ρ.

### 4.3. Simulation and Results

To prove the effectiveness of Algorithm 2 and consider the dependence of the result on the λ parameter, we conducted the simulation as follows. From a set of chosen points $P=\{p_{1},p_{2},\ldots ,p_{r}\}$ representing the anomaly positions, the simulation program generated the test dataset this way: from each point $p_{i}$, create *k* points $P_{i}=\left\{p_{i1},p_{i2},\ldots ,p_{ik}\right\}$ that represent the observations reported for anomaly *i*. Points $p_{ij}$ are randomly generated according to the Gaussian distribution with variance $\sigma_{xij}^{2}=\sigma_{yij}^{2}=\sigma_{ij}^{2}$ around point $p_{i}$. The value of $\sigma_{ij}$ is also a random value between $\sigma_{min}$ and $\sigma_{max}$. In these simulations, $\sigma_{min}$ was set to 2.12 and $\sigma_{max}$ was set to 8.48, which corresponds to a GPS accuracy between 3 m and 12 m, respectively.

The set of k×r generated points served as the test data. The list of obtained maximum points was compared with the original set of points *P* to evaluate the results.

During the experiment, we first observed the effect of the parameter on the results in order to select the parameters before proceeding to obtain the final results. We experimented with the case of one mode, λ=0.9, ε=0.01, with the respective number of points of 20, 50, 100, and 1000. The program-produced convergence points with the average distance and the standard deviation, calculated over 1000 tests, were respectively (0.008, 0.0123), (0.012, 0.0147), (0.014, 0.0154), and (0.013, 0.0078). We choose the parameter ρ=0.1, which is a sufficiently large value, to ensure that these points were only counted for one mode. On the other hand, this value was very small compared to the GPS error. Hence, we chose ε=0.01 and ρ=0.1 for the experiments.

Our experiments were conducted on a set of anomalies *P* consisting of 10 points of imaginary anomalies. The center of the coordinate system was at point (lat=48.817456,lon=2.419799). The vertical axis was in the south-north direction. The horizontal axis was directed from west to east. The length unit was meters. The ten points were at coordinates (−23,0), (−28,0), (−8,0), (0,0), (15,0), (25,0), (37,0), (0,35), (−5,20), and (10,22).

**Figure 5:**
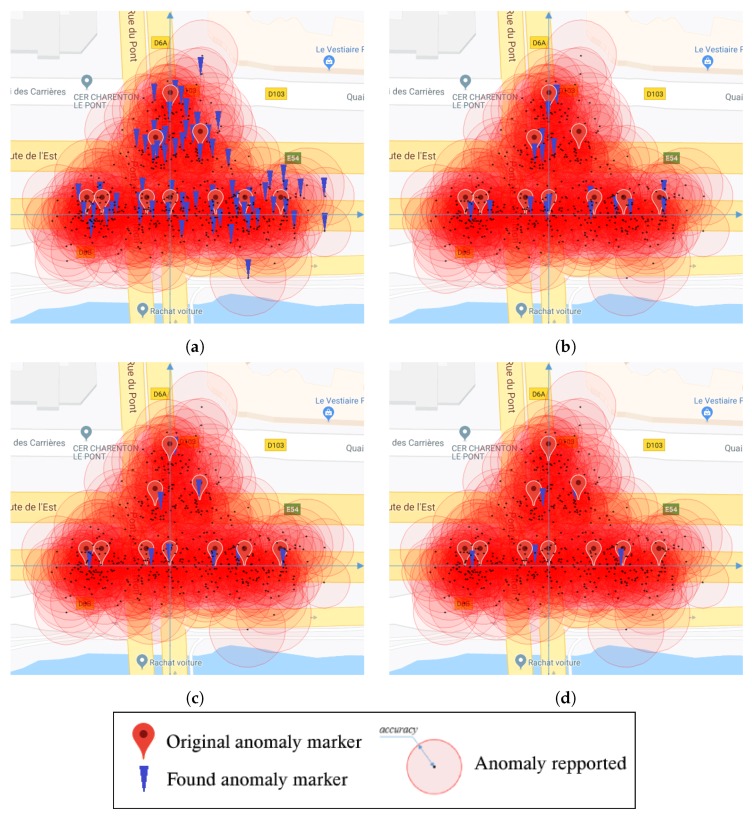
Visual results of the simulation. (**a**) λ=0.4; (**b**) λ=0.6; (**c**) λ=0.9; (**d**) λ=1.6.

In the first experiment, we studied the results with different values for λ. For each of the ten original points in *P*, we generated *k* = 50 data points. Thus, the cluster reported 500 anomalies. Experimental results on this dataset, with the corresponding parameters λ, were 0.4, 0.6, 0.9, and 1.6, respectively, as shown in Figure 5. These results showed that when the bandwidth was too small, the mean shift algorithm obtained too many maxima points. Then, the larger the bandwidth, the smaller the number of maxima points as the nearest maxima points gradually became one. With the appropriate λ values, e.g., λ=0.9 in the above experiment, the results were very good, except for two very close points. In the above experiments, they were located at a 5-m distance, and the program only identified them as a single point located in the middle.

We conducted the next experiment to evaluate quantitatively the dependence of the algorithm results on λ. First, we need to define an objective function. Assume *P* is the set of initial anomalies, *Q* is the set of modes obtained from the algorithm, p∈P, and q∈Q. Let:

$$\mathrm{found}\left(p,Q\right)=\left\{\;\begin{array}{cc} \mathrm{true} & \mathrm{if}\;\exists \;q^{*}\in \mathrm{such}\;\mathrm{that}∥p-q^{*}∥<\omega \\ \mathrm{false} & \mathrm{otherwise} \end{array}\right.$$

$$\mathrm{matched}\left(q,P\right)=\left\{\;\begin{array}{cc} \mathrm{true} & \mathrm{if}\;\exists \;p^{*}\in P\mathrm{such}\;\mathrm{that}∥p^{*}-q∥<\omega \\ \mathrm{false} & \mathrm{otherwise} \end{array}\right.$$

Then, objective function *T* can be defined as:

T(P,Q)=|{p:p∈P∧found(p,Q)}|−|{q:q∈Q∧¬matched(q,P)}|

We chose ω = 4 m for these experiments. We conducted experiments with sets of two points and determined that, if the distance between the two points was less than 4 m, the probability of identifying both anomalies was less than 10%.

**Figure 6:**
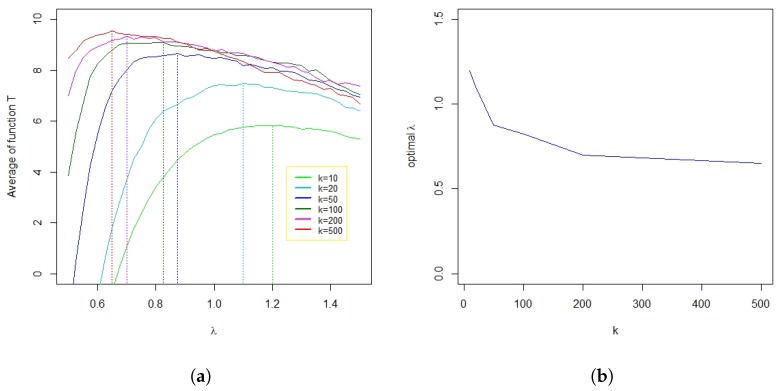
Objective function and optimal λ. (**a**) Average of the objective function according to λ; (**b**) optimal value of λ according to k.

Figure 6 shows the results of the experiments for the 10 original points above with six different values for *k*. For each value of *k*, the test program generated 100 datasets, each of which was used to test each value of λ from 0.5–1.5 (with steps of 0.025). The graphs allowed us to draw some of the following properties:

- In general, the larger the number of data, the better the results. With k≥50, the result of the program was very good, i.e., close to 10 (as data were generated with 10 maxima to find).
- The larger the dataset, the smaller the required λ value for the algorithm to achieve the optimal result (see Figure 6b).
- More experiments on real data were needed to estimate good λ values as a function of the total number of data points *n*. According to past experiments, λ should be chosen in the range from 0.7–0.9. Since this value is quite small, the program usually results in identifying more anomalies than there are effectively, especially when the size of the dataset is small. However, the program tends to eliminate weighted points below a given threshold. This not only eliminates cases where there are few reported data, but it also allows the removal of small maxima points, often located far from real anomalies.

## 5. Conclusions and Future Work

In this article, we proposed an architecture to detect road anomalies using a mobile phone sensor system. For this model, we focused on building lightweight algorithms to be used on smartphones to detect anomalies and on building algorithms to aggregate anomaly data on the server.

For the anomaly detection, we proposed to apply the Grubbs test to improve the classic threshold-based methods chosen for their lightweight and adaptive real-time approach. Experiments for road anomaly detection on real data collected by both cars and scooters suggested that the method we proposed gave good results and was well adapted to different types of vehicles.

Regarding the anomaly data aggregation, we proposed a two-stage method. First, reported anomaly data were divided into separate clusters. Then, a mean shift-based algorithm was applied to find modes, i.e., the most likely position of the anomalies. Simulations showed very good results. This experiment also allowed analyzing how to select the appropriate parameters according to the data characteristics.

In the future, we aim at improving the model by applying a machine learning method in the server to increase the accuracy of the anomaly detection and classify them. We will also do experiments on real data to validate the anomaly data aggregation method. 

## Figures and Tables

**Table 1:** Comparison values of the algorithms, © 2019 IEEE [4].

Algorithm	Value $X_{i}$	Anomaly Condition
Z-THRESH	$a_{z}^{last}$	$X_{i}>\bar{X}+\sigma *G_{N,\alpha }$ *or* $X_{i}<\bar{X}-\sigma *G_{N,\alpha }$
Z-DIFF	$|a_{z}^{last}-a_{z}^{last-1}|$	$X_{i}>\bar{X}+\sigma *G_{N,\alpha }$
STDDEV(Z)	$stddev(a_{z}^{i-winSize}\ldots a_{z}^{last})$	$X_{i}>\bar{X}+\sigma *G_{N,\alpha }$
G-ZERO	$max(a_{x}^{last},a_{y}^{last},a_{z}^{last})$	$X_{i}<\bar{X}-\sigma *G_{N,\alpha }$
DVA-THRESH	${\left(a_{z\_max}-a_{z\_min}\right)}^{1sec}$	$X_{i}>\bar{X}+\sigma *G_{N,\alpha }$

## Abbreviations

The following abbreviations are used in this manuscript:

SVM	Support Vector Machines
FFT	Fast Fourier Transformation
ROC	Receiver Operating Characteristic curve
TP	True Positive
FP	False Positive
TN	True Negative
FN	False Negative
